# Effect of antioxidants on BPA-induced stress on sperm function in a mouse model

**DOI:** 10.1038/s41598-019-47158-9

**Published:** 2019-07-22

**Authors:** Md Saidur Rahman, Kyu-Ho Kang, Sarder Arifuzzaman, Won-Ki Pang, Do-Yeal Ryu, Won-Hee Song, Yoo-Jin Park, Myung-Geol Pang

**Affiliations:** Department of Animal Science & Technology and BET Research Institute, Chung-Ang University, Anseong, Gyeonggi-do 456-756 Republic of Korea

**Keywords:** Infertility, Molecular medicine, Infertility, Molecular medicine

## Abstract

In the past few years, bisphenol A, (BPA) an endocrine-disrupting chemical, has received increasing attention because of its detrimental health effects. There is ample evidence to support that BPA interferes with the reproductive health of humans and animals. In spermatozoa, BPA-induced adverse effects are mostly caused by increased oxidative stress. Using an *in vitro* experimental model, we examined whether antioxidants (glutathione, vitamin C, and vitamin E) have defensive effects against BPA-induced stress in spermatozoa. The results showed that antioxidants inhibit the overproduction of reactive oxygen species (basically cellular peroxides) and increase intracellular ATP levels, thereby preventing motility loss and abnormal acrosome reaction in BPA-exposed spermatozoa. In particular, glutathione and vitamin E reduced the protein kinase A-dependent tyrosine phosphorylation in spermatozoa and, thus, prevented the precocious acrosome reaction from occurring. Furthermore, we found that the compromised fertilisation and early embryo development mediated by BPA-exposed spermatozoa can be improved following their supplementation with glutathione and vitamin E. Based on these findings, we suggest that antioxidants reduce oxidative stress in BPA-exposed spermatozoa, thus preventing detrimental effects on their function and fertility.

## Introduction

Bisphenol A [BPA; 2,2-bis(4-hydroxyphenyl) propane] is a common endocrine disruptor that is widely used in plastic industries and is capable of altering the synthesis, metabolism, transport, and elimination processes of endogenous hormones and, thus, of mimicking/antagonizing hormonal activities in the body^1^. Recent studies have suggested that BPA causes developmental, systemic, neurological, immune, and reproductive disorders^2,3^. As a reproductive toxicant, it is linked with impaired steroidogenesis, defective embryo development, testicular/epididymal malfunction, abnormal accessory sex gland functions, and so on, and thus causes subfertility/infertility^2,4,5^. The easiest way to avoid BPA toxicity is to minimise its exposure. However, human exposure to this chemical is ubiquitous via oral, respiratory, and dermal routes^2,6,7^, and it is practically challenging to limit its use in everyday life. Therefore, the development of potential therapeutic strategies is one of the best options for minimizing BPA toxicity.

BPA acts as a selective estrogen receptor (ER) modulator by binding with genomic (nuclear and cytoplasmic) and non-genomic (membrane bound) ERs in cells^2,7^. In addition, BPA acts as an activator of estrogen-related receptor gamma^8^ and growth factor receptors^9^, antagonises thyroid hormone receptor^10^, and possesses anti-androgenic properties^11^. Mature spermatozoa contain the majority of these receptors (e.g. ERα, ERβ, growth factor, and androgen receptor)^12^; therefore, spermatozoa are potentially a suitable model for examining the effects and molecular mechanism of BPA, as well as its subsequent effects on male fertility/reproduction.

Recently, we showed that the exposure of mice spermatozoa to 100 µM BPA exhibits a rapid non-genomic estrogenic signaling by the activation of several kinase systems^7^. Decreased motility, abnormal acrosome reaction, altered mitochondrial activities, and decreased fertilisation and embryo development were observed in the BPA-exposed spermatozoa^6,7^. Similar adverse effects have also been reported in F1 spermatozoa following gestational exposure to BPA^13,14^. These harmful effects appeared mostly because of the increased oxidative stress in BPA-treated spermatozoa as detected by elevated reactive oxygen species (ROS) levels^6,13,14^. Furthermore, a few studies have reported an increase in BPA-induced oxidative stress in neuronal^15^ and kidney cells^16^. Although low levels of ROS are important for regulating normal sperm functions, such as capacitation^17^, acrosome reaction^18^, and motility hyper-activation^19^, excessive ROS production adversely affects the lipid, protein, and DNA structure in the sperm, subsequently causing infertility^17^.

Antioxidants are molecules that inhibit oxidation and thereby prevent oxidative stress or overproduction of ROS in cells and tissues. Previous studies have shown that antioxidants reduce oxidative stress in spermatozoa during cryopreservation and exert positive effects on sperm motility and fertilisation potential^20–22^. However, no studies have investigated the protective effects of antioxidants on BPA-exposed spermatozoa thus far. Therefore, in the current study, the above-mentioned findings were used to examine whether the supplementation of commonly used antioxidants, such as glutathione (GSH; gamma-glutamylcysteinylglycine), vitamin C (Vit C; L-ascorbic acid), and vitamin E (Vit E; α-tocopherol), has positive effects on the function of BPA-exposed spermatozoa.

## Results

### Effect of antioxidants on motility parameters of BPA-exposed spermatozoa

First, we evaluated whether antioxidants defended motility parameters in BPA-exposed spermatozoa. We noticed that an exposure to 100 µM BPA significantly decreased the percentage of motile spermatozoa compared to the control (*P* < 0.01). However, the motility of the BPA-exposed spermatozoa supplemented with antioxidants, was significantly higher than that of the BPA only group (*P* < 0.01). Additionally, there was a complete recovery of the reduced dance characteristics in the GSH and Vit E co-exposure groups (*P* < 0.05), but not in the Vit C-treated group, as noticed in the BPA-exposed spermatozoa. Unlike overall motility, we observed minor variations in the curvilinear (VCL), straight-line (VSL), and average path velocities (VAP); however, the hyperactivated motility (HYP) and other kinematic parameters of spermatozoa were potentially unchanged between the control and treatment groups (Table 1).

**Table 1:** Changes in sperm motility parameters following exposure to either BPA or BPA with antioxidants compared to the control.

Parameters	Control	BPA	BPA + GSH	BPA + Vit C	BPA + Vit E	*P*-value
MOT (%)	81.54 ± 0.88^a^	51.39 ± 1.91^b^	64.51 ± 1.64^c^	66.55 ± 2.22^c^	67.40 ± 3.11^c^	<0.001
HYP (%)	9.66 ± 0.87	8.35 ± 0.37	11.31 ± 1.07	8.55 ± 1.23	8.93 ± 1.36	Ns
VCL (µm/s)	113.90 ± 3.61^a,b^	113.38 ± 3.22^a,b^	122.81 ± 3.30^b^	102.93 ± 2.20^a^	115.23 ± 3.69^b^	0.005
VSL (µm/s)	42.82 ± 1.10^a^	39.45 ± 2.00^a,b^	43.64 ± 0.98^a^	37.09 ± 1.24^b^	41.32 ± 1.2^a,b^	0.023
VAP (µm/s)	54.89 ± 2.27^a^	51.22 ± 1.34^a,b^	55.44 ± 1.29^a^	46.76 ± 1.40^b^	53.91 ± 1.05^a^	0.003
LIN (%)	38.09 ± 1.54	36.57 ± 1.25	35.93 ± 0.37	37.91 ± 1.19	35.88 ± 0.33	Ns
BCF	13.57 ± 0.54	12.91 ± 0.37	12.38 ± 0.23	13.57 ± 0.31	13.18 ± 0.24	Ns
WOB	48.36 ± 0.88	46.08 ± 0.39	45.55 ± 0.69	47.73 ± 0.75	46.89 ± 0.85	Ns
DNC	578.37 ± 15.15^a^	444.78 ± 13.48^b^	640.2 ± 11.12^a^	421.41 ± 9.86^b^	585.74 ± 8.2^a^	0.009
DNM (µm)	13.37 ± 1.23	13.24 ± 0.59	14.63 ± 0.48	11.35 ± 0.51	13.13 ± 0.42	Ns
ALH (µm)	4.96 ± 0.32	4.78 ± 0.14	5.24 ± 0.13	4.29 ± 0.12	5.07 ± 0.13	Ns

All data were analysed by one-way analysis of variance (ANOVA). Tukey’s test was used to identify differences between groups. MOT = motility, HYP = hyperactivated motility, VCL = curvilinear velocity, VSL = straight-line velocity, VAP = average path velocity, LIN = linearity, BCF = beat cross frequency; WOB = wobble, DNC = dance, DNM = dance mean, ALH = mean amplitude of head lateral displacement, Ns = not significant, GSH = gamma-glutamylcysteinylglycine, Vit C = vitamin C, Vit E = vitamin E. Data are the means ± SEM of six experimental replicates (three mice per replicate). Values with different superscript characters (^a,b,c^) indicate significant differences between the control and treatment groups as determined by one-way analysis of variance. Tukey’s test was used to identify differences between groups. The concentration of bisphenol A (BPA), glutathione (GSH), vitamin C (Vit C), and vitamin E (Vit E) were 100 µM, 5 mM, 100 µM, and 2 mM, respectively.

### Effect of antioxidants on the capacitation status of BPA-exposed spermatozoa

**Figure 1:**
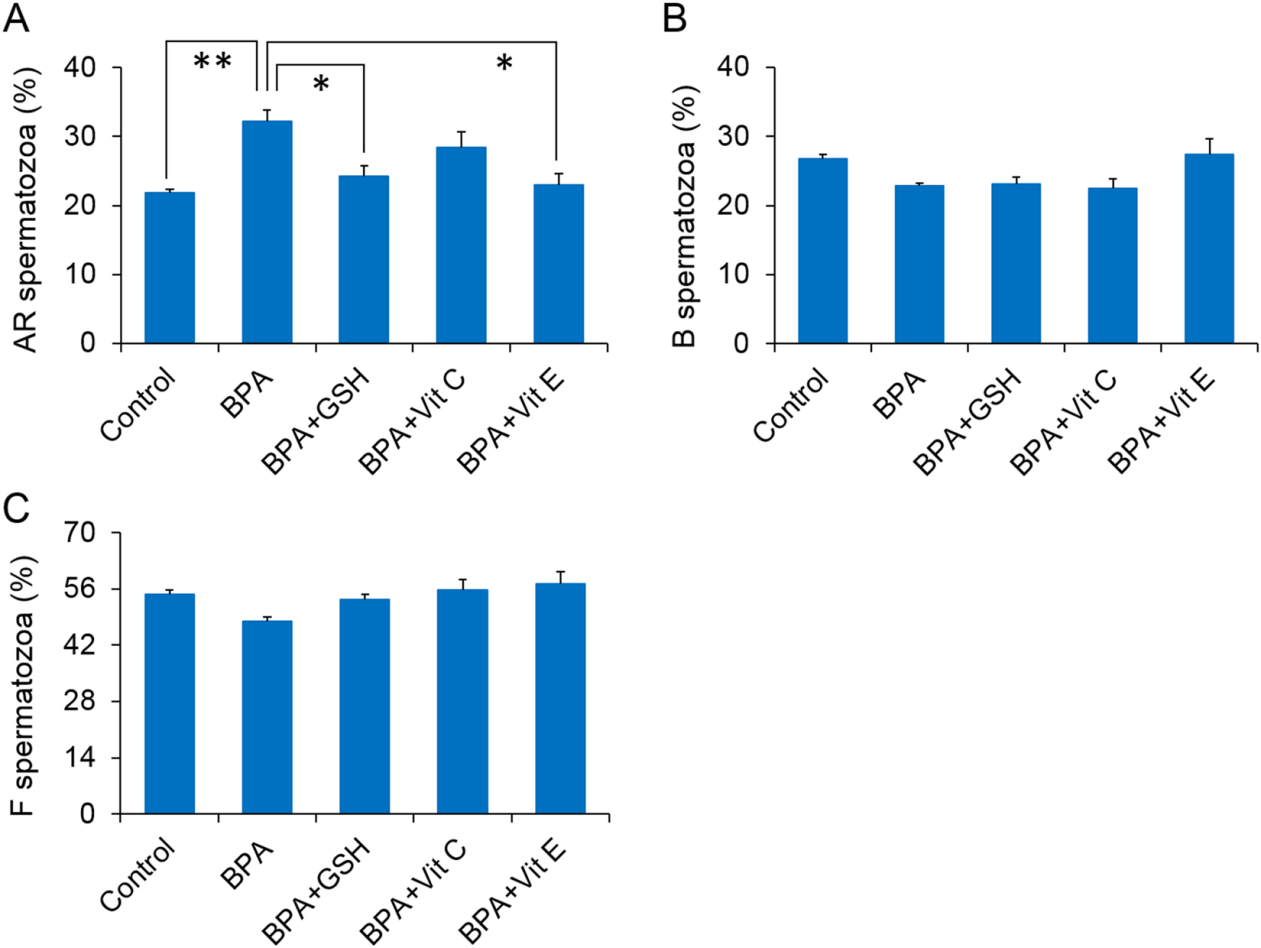
Changes in sperm capacitation status in control and treated spermatozoa. (**A**) Percentage of the acrosome-reacted (AR spermatozoa) spermatozoa. (**B**) Percentage of the capacitated (B spermatozoa) spermatozoa. (**C**) Percentage of the non-capacitated (F spermatozoa) spermatozoa. The data presented are the means of four experimental replicates with three mice per replicate. Data are show as means ± SEM. All data were analysed using one-way analysis of variance. Tukey’s test was used to identify differences between groups. *P < 0.05; **P < 0.01. The concentration of bisphenol A (BPA), glutathione (GSH), vitamin C (Vit C), and vitamin E (Vit E) were 100 µM, 5 mM, 100 µM, and 2 mM, respectively.

We evaluated the changes in the sperm capacitation status following exposure to BPA alone and with antioxidants. As shown in Fig. 1A, the number of acrosome-reacted spermatozoa (AR spermatozoa) was significantly increased following *in vitro* exposure to BPA only, compared to the control conditions (*P* < 0.01). When BPA-exposed spermatozoa were additionally supplemented with GSH and Vit E, the number of AR spermatozoa were comparable to the control level (*P* < 0.05), unlike when they were supplemented with Vit C. In addition, no changes were observed in the number of capacitated (B spermatozoa) and non-capacitated spermatozoa (F spermatozoa) following exposure to BPA alone and with antioxidants (Fig. 1B,C).

### Effect of antioxidants on protein kinase-A (PKA) and tyrosine phosphorylation of BPA-exposed spermatozoa

**Figure 2:**
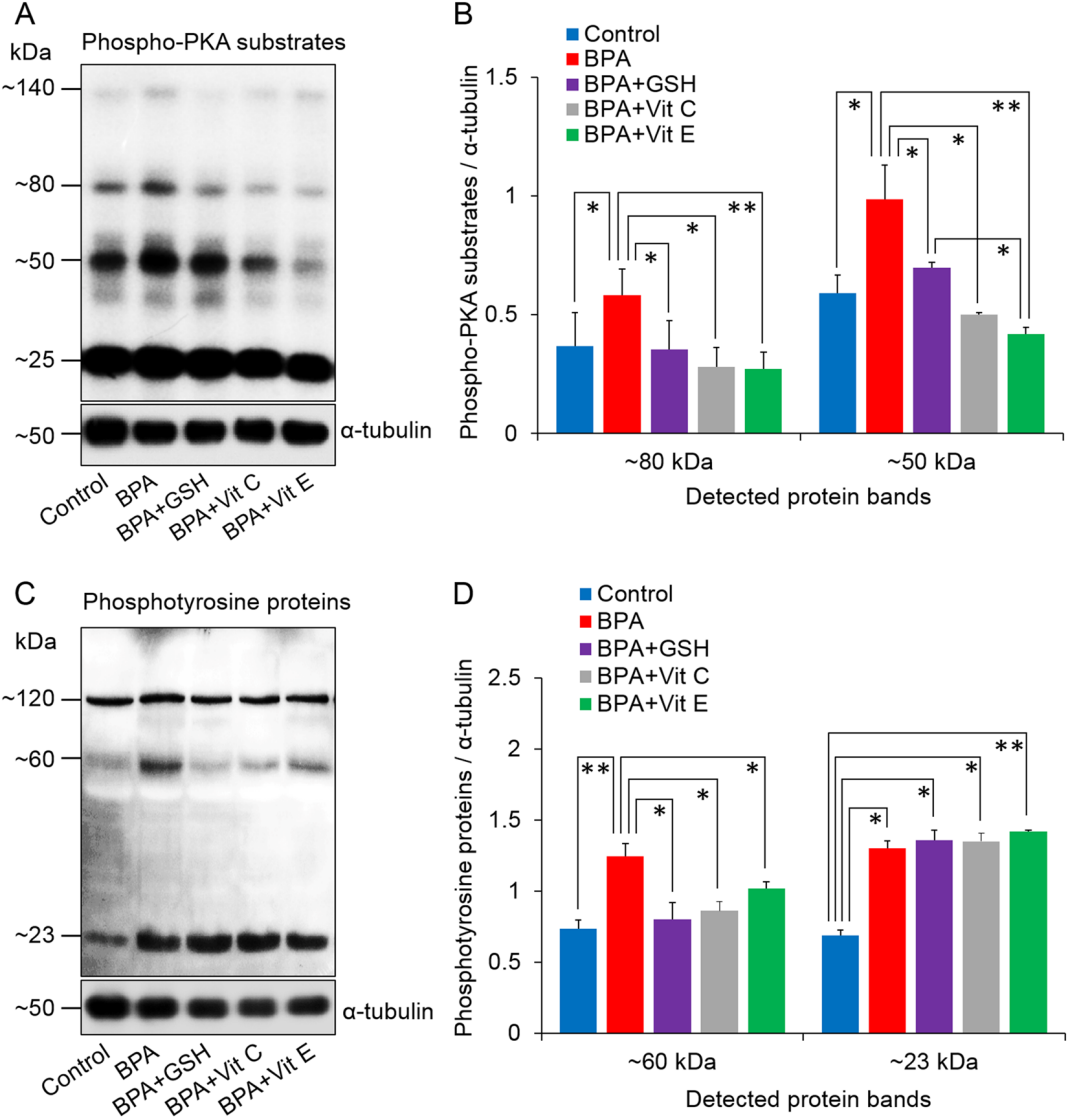
Changes in protein kinase A (PKA) activity and tyrosine phosphorylation levels in spermatozoa. (**A**) Representative western blot image of phospho-PKA substrates. (**B**) Density of phospho-PKA substrates. (**C**) Representative western blot image of tyrosine phosphorylated proteins. (**D**) Density of tyrosine phosphorylated proteins. The data presented are the means ± SEM of three replicate experiments with three mice per replicate. All data were analysed using one-way analysis of variance. Tukey’s test was used to identify differences between treatments. *P < 0.05; **P < 0.01. Representative uncropped immunoblots are shown in Supplementary Fig. S2. The concentration of bisphenol A (BPA), glutathione (GSH), vitamin C (Vit C), and vitamin E (Vit E) were 100 µM, 5 mM, 100 µM, and 2 mM, respectively.

We also evaluated the influence of BPA alone and with antioxidants on the PKA and protein tyrosine phosphorylation activities in spermatozoa. As supported by earlier studies^6,23^, we observed that the PKA activity in spermatozoa was elevated (both in ~80 and ~50 kDa) following exposure to 100 µM BPA, compared to PKA activity measured in control cells (*P* < 0.05). The PKA activities of the spermatozoa exposed to BPA with antioxidants, were comparable to the control level (Fig. 2A,B). Similar changing patterns were noticed in protein tyrosine phosphorylation levels in ~60 kDa following exposure to either BPA alone or with antioxidants. In addition, we noticed significantly higher protein tyrosine phosphorylation in ~23 kDa for all exposure conditions than that in the control spermatozoa (*P* < 0.05) (Fig. 2C,D).

### Effect of antioxidants on intracellular ATP levels of BPA-exposed spermatozoa

**Figure 3:**
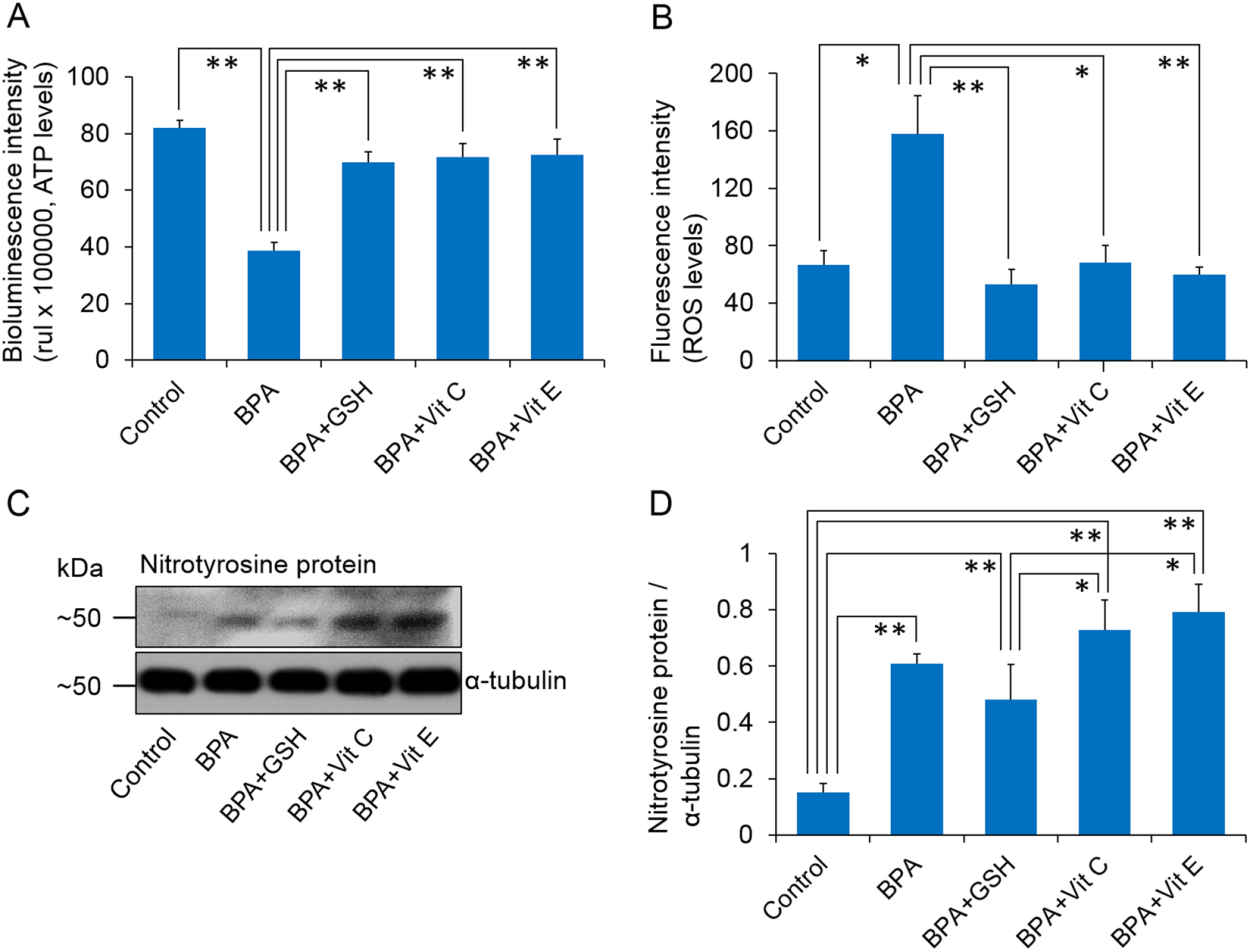
Levels of intracellular ATP, reactive oxygen species (ROS), and nitro tyrosine proteins in control and treated spermatozoa. (**A**) Bioluminescence intensities (proportional to the intracellular ATP levels) of the control and treated spermatozoa. (**B**) Fluorescence intensities (proportional to the intracellular ROS levels) of the control and treated spermatozoa. (**C**) Representative western blot image of nitro tyrosine proteins. (**D**) Density of nitro tyrosine proteins. The data presented are the means ± SEM of three replicate experiments (three mice per replicate). All data were analysed using one-way analysis of variance. Tukey’s test was used to identify differences between groups. *P < 0.05; **P < 0.01. Representative uncropped immunoblots are shown in Supplementary Fig. S3. The concentration of bisphenol A (BPA), glutathione (GSH), vitamin C (Vit C), and vitamin E (Vit E) were 100 µM, 5 mM, 100 µM, and 2 mM, respectively.

Further, we sought to understand whether antioxidants sustain cellular ATP levels in the BPA-exposed spermatozoa. We noticed significantly lower ATP levels in only BPA-exposed spermatozoa than those in the control and other groups (*P* < 0.01). Simultaneously, the ATP levels were similar between the antioxidant-supplemented and control groups (Fig. 3A).

### Effect of antioxidants on intracellular ROS and protein tyrosine nitration levels of BPA-exposed spermatozoa

To elucidate the stress response mechanisms in spermatozoa following different treatments, we evaluated the intracellular ROS (basically cellular peroxides) and protein tyrosine nitration levels. We observed significantly higher ROS levels in BPA-exposed spermatozoa than in the control and other groups (*P* < 0.05; Fig. 3B). Simultaneously, we noticed the complete recovery of oxidative stress induced by increased ROS in BPA-exposed spermatozoa following co-exposure with antioxidants. Conversely, the groups treated with BPA alone and with antioxidants showed significantly (*P* < 0.01) higher protein tyrosine nitration than the control group (Fig. 3C,D). In addition, the groups treated with BPA + GSH showed lower tyrosine nitration than the group treated with BPA + Vit C and BPA + Vit E (*P* < 0.05; Fig. 3C,D).

### Effect of antioxidants on fertilisation and early embryo developments ability of BPA-exposed spermatozoa

**Figure 4:**
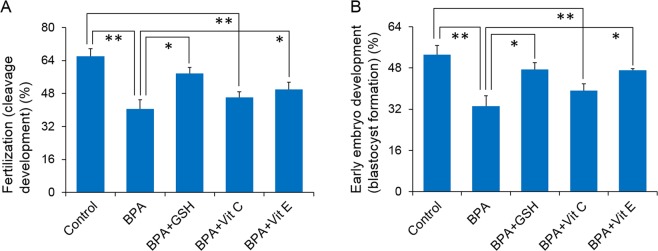
Percentage of fertilisation and embryo development by the control and treated spermatozoa. (**A**) Percentage of cleavage (fertilisation) development. (**B**) Percentage of blastocyst (early embryonic development) formation. The data presented are the means ± SEM of four replicate experiments (three male and three female mice per replicate). All data were analysed using one-way analysis of variance. Tukey’s test was used to identify differences between groups. *P < 0.05; **P < 0.01. The concentration of bisphenol A (BPA), glutathione (GSH), vitamin C (Vit C), and vitamin E (Vit E) were 100 µM, 5 mM, 100 µM, and 2 mM, respectively.

Finally, we examined the ability of spermatozoa to fertilise an oocyte and undergo early embryonic development using an *in vitro* fertilisation (IVF) assay. Particularly, we wanted to understand whether antioxidants could protect the fertilising ability of spermatozoa after BPA exposure. We demonstrated that both GSH and Vit E were capable of restoring the fertilisation and early embryo development capabilities of BPA-exposed spermatozoa (*P* < 0.05; Fig. 4). Vit C slightly increased the fertilisation and embryonic development potential of BPA-exposed spermatozoa; however, the increase was statistically non-significant (Fig. 4A,B).

## Discussion

BPA is a high-production-volume endocrine disruptor chemical that can interfere with the reproductive health of both humans and animals. The adverse effects of BPA on cells/tissues are mostly contributed via increased oxidative stress^6,7,24^; thus, we assumed that antioxidants could be effective therapeutic targets to overcome BPA toxicity. In the present study, we chose the mouse sperm model because mice have a high phenotypic/physiological similarity with humans^25^. In addition, spermatozoa contain specific receptors/transporter for both BPA and selected antioxidants^26–28^, and, thus, were an excellent model to confirm our hypothesis.

Sperm motility is an essential predictor of male fertility, and optimum sperm motility and motion kinematics are a benchmark of successful fertilisation^29^. It has been demonstrated that the supplementation of antioxidants produce protective effects on semen quality, including vitality, motility, and morphology of spermatozoa^30^. Considering an *in vitro* experimental model, the beneficial effects of antioxidants, including GSH, Vit C, and Vit E, on sperm function and fertilizing ability also have been reported^31–33^. In agreement with previous findings, the results from the present study demonstrated that BPA-induced decreases in sperm motility were mostly recovered by antioxidants. In addition, elevated oxidative stress (measured as ROS levels) and decreased intracellular ATP levels in BPA-exposed spermatozoa were completely recovered by antioxidants. Increased ROS and their metabolites are major culprits targeting DNA, lipids, proteins, and enzymatic systems of spermatozoa, leading to an irreversible injury to cells, and a decline in motility and fertilizing competence^17,34^. Therefore, one possibility for this effect could be that the antioxidants prevented excessive stress in BPA-exposed spermatozoa, subsequently maintaining sperm motility. In addition, we recently showed that an exposure to 100 µM BPA was capable of altering mitochondrial activity and subsequently declining cellular ATP levels that support motility in mice spermatozoa^7^. Physiologically, mitochondria regulate a series of key cellular events, such as ATP and ROS generation, detoxification, and apoptosis^35^. Mitochondrial ROS must be maintained at optimal concentrations in order to balance their delicate interaction with intracellular antioxidants^35^. Therefore, another possible effect of antioxidants on the motility of spermatozoa is that antioxidants might improve their ability to generate ATP as noted in the current study, subsequently support motility in BPA-treated microenvironments.

Conversely, oxidative stress, generated by elevated ROS levels, is predisposed to redox-dependent protein modifications, such as tyrosine nitration, in spermatozoa^36,37^. An earlier study showed that gestational exposure to BPA increased the levels of protein tyrosine nitration in F1 capacitated spermatozoa in adulthood, subsequently affecting fertility^14^. In the present study, we demonstrated that although antioxidants completely defused ROS levels, higher tyrosine nitration was noticed in the BPA-only and all BPA with antioxidants groups than that in the control group. The DCFDA-cellular ROS detection assay used in the present study was based on the cell permeant reagent 2′,7′ dichlorofluorescin diacetate dye that is capable of measuring basically hydroxyl and peroxyl, and other ROS activity within cells. However, tyrosine nitration is induced by peroxynitrite (ONOO^−^)^38^. Therefore, we assumed that although antioxidants could neutralize total ROS in BPA-exposed spermatozoa, spermatozoa were incapable of overcoming the ONOO^−^ -mediated stress completely. However, further studies are necessary in order to support this hypothesis.

For fertilisation, mammalian spermatozoa must undergo a series of physiological and biochemical modifications in the female reproductive tract, collectively known as capacitation^39,40^. Capacitated spermatozoa subsequently undergo hyper-activation and acrosome reaction to penetrate the zona pellucida and fuse with the oocyte^39–42^. At the molecular level, both capacitation and acrosome reaction are associated with increased fluidity of the sperm membrane, adjustment of the intracellular ionic equilibrium, and activation of soluble adenylyl cyclase, cAMP, and PKA^39,42^. Further, the activated PKA phosphorylates several capacitation-related target proteins in spermatozoa, mostly at the tyrosine residues^39,40,42^. There is ample evidence to support that tyrosine phosphorylation during capacitation is controlled by the activity of cAMP/PKA^6,7,14,39^. In the present study, we observed that in BPA-exposed spermatozoa co-incubated with GSH or Vit E, the number of AR cells returned to the control level. Consistently, the increased PKA activity (both in ~80 and ~50 kDa) and tyrosine phosphorylation (in ~60 kDa) also returned to the baseline. The effect of BPA together with PKA and protein tyrosine phosphorylation on the acrosome reaction has been evidenced in previous studies^6,7^. Wang *et al*.^23^ reported that BPA was capable of stimulating PKA and protein tyrosine phosphorylation in rat spermatozoa, with even the sperm culture media being devoid of capacitation inducing factors (e.g. BSA, HCO_3_^−^, and Ca^2+^). ROS is involved in the regulation of the acrosome reaction^43^; therefore, the increased ROS levels in BPA-exposed spermatozoa might be responsible for the resulting acrosome reaction. In support of our hypothesis, extracellular ROS (O_2_^−^ and H_2_O_2_) in association with tyrosine phosphorylation have been reported as inducer of the acrosome reaction in human spermatozoa^18^.

Although the effect of antioxidants on capacitation/acrosome reaction is not justified, it has been reported that the addition of antioxidants, including GSH, Vit C, and Vit E, during cryopreservation prevented motility loss in freeze-thawed bull, ram, and goat spermatozoa by improving their viability, chromatin assembly, and membrane integrity^44^. Similar effects of antioxidants have been shown in boar spermatozoa during liquid preservation^45^. Antioxidants are capable of breaking the oxidative chain reaction in cells, for example, Vit E directly diminishes free radicals, particularly peroxyl and alkoxyl (ROO•), produced through ferrous ascorbate-induced lipid peroxidation^44^, thus minimizing the intracellular oxidative stress and subsequently restoring the acrosomal membrane integrity. Therefore, we suggest that the increased acrosome reaction following exposure to BPA might reflect the BPA-induced loss of acrosome integrity by controlling the PKA-dependent phosphorylation of tyrosine residues in spermatozoa. Antioxidants minimise the BPA-induced stress by altering the detrimental consequences, thereby protecting acrosome integrity or preventing the precocious acrosome reaction from occurring.

Another novel finding in the present study was the significant protective effects of antioxidants, i.e. GSH and Vit E, on the fertilisation and embryo development of BPA-exposed cells. In previous studies, we showed that the loss of sperm fertility following *in vitro* exposure to BPA (100 µM) was mediated by decreased motility and premature triggering of the acrosome reaction^6,7^. Decreased motility and abnormal capacitation have also been reported as leading causes of fertility loss in F1 male mice following gestational exposure to BPA (50 mg/kg bw/day)^13,14^. Under normal circumstances, progesterone secreted by cumulus cells of an unfertilised egg promotes the acrosome reaction in spermatozoa before fertilisation^6,46^. Therefore, if the acrosome reaction occurred in spermatozoa before meeting with the oocyte, these spermatozoa lose their ability to fertilise^6^. Because premature acrosome reaction is associated with altered mitochondrial activity and increased chromatin decondensation associated with oxidative stress^47,48^, it could be prevented via antioxidants. Therefore, we suggest that antioxidants play a protective role against BPA-induced toxicity by reducing the oxidative stress, thus preventing acrosome integrity/premature acrosome reaction and subsequently restoring sperm fertility. On the other hand, antioxidants protect the loss of motility in BPA-exposed spermatozoa, thus improving fertility. In the present study, although GSH and Vit E were found effective in protecting sperm fertility, Vit C was found to be non-responsive. This is possibly because 100 µM of Vit C was incapable of protecting acrosome integrity in BPA-exposed spermatozoa. Therefore, the selection of a more appropriate dose of Vit C should be considered in future studies.

## Conclusions

Collectively, we found that the BPA-induced loss of sperm motility, intracellular ATP, and accelerated abnormal acrosome reaction resulted in compromised fertilisation and early embryo development. These effects were mostly caused by increased oxidative stress induced by BPA. As antioxidants minimise ROS production, they prevented the detrimental consequences of BPA exposure, thereby restoring the fertilisation ability of spermatozoa. The present study has both theoretical and clinical importance because the findings of this study are an initial step toward developing potential therapeutic strategies for the management of BPA toxicity. However, further studies considering *in vivo* experimental design are required to confirm our initial hypothesis.

## Methods

### Ethical statements and animal care

The ethical approval for this animal study was obtained from the Institutional Animal Care and Use Committee of Chung-Ang University, Seoul, Korea (IACUC Number: 2016−00009). All methods were performed in accordance with the relevant guidelines and regulations. Eight-week-old CD-1 (ICR) male mice were purchased from Daehan BioLink^®^ (Eumseong, Chungcheongbuk-do, Korea) and were allowed to adapt for at least 1 week before sample collection. The mice were kept in a room maintained at 20–25 °C, 50–60% humidity, and 12 h day/12 h night conditions. Commercial mouse pellets (Young Bio, Gyeonggi-do, Korea) and water from glass bottles were provided ad libitum during the study period. Maximum precautions were taken in the animal room facilities to minimise background BPA exposure.

### Chemicals and medium

All chemicals and reagents were purchased from Sigma-Aldrich (St. Louis, MO, USA), unless otherwise mentioned. Modified Tyrode’s medium (97.84 mM NaCl, 1.42 mM KCl, 0.47 mM MgCl_2_.H_2_O, 0.36 mM NaH_2_PO_4_H_2_O, 5.56 mM _D_-glucose, 25 mM NaHCO_3_, 1.78 mM CaCl_2_H_2_O, 24.9 mM Na-lactate, and 50 µg/mL gentamycin) was used as the basic media (BM) for spermatozoa. The BM was supplemented with 0.4% bovine serum albumin (BSA) for inducing capacitation and was preincubated at 37 °C in the presence of 5% CO_2_ in air^6,13^. The pH and osmolality of the BM were maintained at 7.2 ± 0.2 and 300 ± 20 mOsm/kg, respectively. Both BPA and antioxidants were dissolved in dimethylsulphoxide (DMSO) and added to the required molecular concentration in BM 1 day prior to the treatment of spermatozoa.

### BPA/Antioxidants doses selection and exposure to spermatozoa

We used 100 µM BPA because BPA at this dose has been found to have detrimental effects on sperm function and fertility by previous studies^6,7^. We also observed harmful effects of this dose of BPA on the functional properties of testicular germ cells (e.g. germ cell viability and proliferation) and spermatogonial stem cells under *in vitro* culture conditions^49^. Three commonly used non-enzymatic antioxidants, i.e. GSH, Vit C, and Vit E, were included in the present study because spermatozoa contain specific transporters for these antioxidants^26–28^. For antioxidant dose selection, we first reviewed several *in silico* studies regarding the effects of antioxidants on sperm parameters in different animal species^31,50–52^. Second, the tentative doses for each antioxidant were considered for a preliminary experiment using mouse spermatozoa to determine whether these concentrations had a potential positive effect on sperm motility (see Supplementary Fig. S1). Finally, 5 mM, 100 µM, and 2 mM of GSH, Vit C, and Vit E, respectively, were used as the target concentrations for the present study.

### Sperm collection, preparation, and exposure to antioxidants and BPA

Mice were euthanized using an intraperitoneal injection of avertin (2,2,2-tribromoethanol). The detailed procedure for the preparation of avertin solution and euthanasia were described in a previous study by our group^14^. Spermatozoa were collected from the cauda epididymis of sexually mature male mice (10–12 weeks old). Briefly, the cauda epididymis was collected carefully from each mouse, placed over a clean dry filter paper, and the surrounding fat was removed. The cauda epididymis was then placed in a culture disc containing BM and was pierced using a sterile needle to disperse the spermatozoa. Following pre-intubation (12 min), the sperm suspension was added to the BM (containing 0.4% BSA) supplemented with BPA alone and BPA with different antioxidants. A total of five treatment groups, including the control, BPA, BPA + GSH, BPA + Vit C, and BPA + Vit E, at set doses were used in the present study. The control spermatozoa were treated with DMSO only. For each experimental replicate, the spermatozoa collected from three male mice were pooled and divided into five groups that were incubated in BM under various exposure schemes. The incubation period of 6 h was found to be the minimum effective period for BPA (100 µM)-exposed mice spermatozoa to exhibit detrimental effects on sperm parameters (e.g. motility and viability)^6,7^; therefore, the sperm suspension was incubated for 6 h in BM under the exposure condition. As such all sperm function tests and proteomic analyses were performed in spermatozoa after 6 h of incubation.

### Computer-assisted sperm analysis (CASA)

The motility and motion kinematics of spermatozoa following exposure with different treatments were analysed using the SAIS-PLUS version 10.1 CASA software program (Medical Supply, Seoul, Korea)^6,13,14^. From each treatment group, 10 µL of sperm sample was placed on the preheated stage (37 °C) of a Makler chamber (Makler, Haifa, Israel) and the 10x phase contrast objective was used by the CASA program to analyse the spermatozoa. The settings of this program were affixed as frames were acquired as follows: frame rate: 30 Hz, minimum contrast: 7, minimum size: 5, low/high size gates: 0.4–1.5, low/high intensity gates: 0.4–1.5, non-motile head size: 16, and non-motile brightness: 14. Five representative fields with at least 250 sperm cells were randomly selected to analyse the percentage of motile/hyperactivated spermatozoa and sperm motion kinematics. For each of the six independent replicate experiments, three male mice were considered for the final results.

### Hoechst 33258/Chlorotetracycline (H33258/CTC) staining

The effects of BPA and antioxidants on the capacitation status of spermatozoa were analysed by dual staining methods using two different fluorescence dyes^6,13^. After incubation, the sperm samples from each treatment group were centrifuged at 100 × *g* for 2.5 min and the supernatant was discarded. Subsequently, 15 µL of H33258 solution was added to 135 µL of sperm sample and maintained at room temperature (20 °C) for 2 min. Thereafter, 2% polyvinylpyrrolidone in phosphate buffer saline (PBS) solution was added and centrifuged at 100 × *g* for 2.5 min. The sperm pellets were resuspended in 100 µL of the CTC solution and 100 µL of PBS. After staining, 10 µL of sperm sample was smeared onto a glass slide with a cover glass. The capacitation status of the sperm samples from each treatment was observed using a Microphot-FXA microscope (Nikon, Tokyo, Japan) under epifluorescence illumination (ultraviolet, BP 340 ± 380/LP 425 with BP 450 ± 490/LP 515 excitation/emission filters).

In accordance with the criteria of Maxwell and Johnson^53^, four different sperm patterns were observed: the D pattern (dead sperm with blue fluorescence on the entire sperm head); the F pattern (live non-capacitated spermatozoa with yellow fluorescence on the entire head); the B pattern (live capacitated spermatozoa with bright yellow fluorescence on the acrosomal region and a dark post-acrosomal region); and the AR patter (live acrosome-reacted spermatozoa with no fluorescence on the sperm head). At least 400 spermatozoa were analysed on each glass slide, and each procedure was repeated at least 3 times for each treatment group. For each of the four independent replicate experiments, three male mice were considered for the final results.

### Detection of intracellular ATP levels

The intracellular ATP levels were measured using an ATP Bioluminescence Assay Kit (CLS II; Roche Molecular Biochemicals, Mannheim, Germany) according to the manufacturer’s protocol^6^. After incubation, sperm concentration was normalised for each exposure condition. The normalised samples were (25 μL) placed in a 96‐well plate together with equal amounts of the lysis reagent and incubated for 5 min at room temperature. Finally, the luciferase reagent (50 μL) was added to the samples in the 96‐well plate, and luminescence was detected using the GloMax®‐Multi Microplate Multimode Reader (Promega, Madison, WI, USA). For each of the three independent replicate experiments, three male mice were considered for the final results.

### Detection of cellular ROS levels

The cellular ROS level was evaluated using a 2′,7′-dichlorofluorescein diacetate (DCFDA) kit (ab113851, Abcam, Cambridge, England)^6,13^. After incubation, we normalised sperm concentration for each exposure condition. The samples were then resuspended in the DCFDA mix and incubated at 37 °C for 30 min. Subsequently, the sperm samples were washed twice with 1 mL of 1X buffer solution and resuspended in 500 µL of 1X supplemental buffer solution. Finally, the sperm suspension was placed in a 96-well plate and the fluorescence was detected using the SoftMax Pro 5 software program (Molecular Devices, San Jose, CA, USA) at the two excitation wavelengths of 485 nm and 535 nm. For each of the three independent replicate experiments, three male mice were considered for the final results.

### Western blotting

Following incubation under different treatment conditions, the spermatozoa (500 × 10^6^ sperm cells/mL) from each treatment group were washed with PBS for 10 min. After discarding supernatants, the sperm pellets were resuspended in the Laemmli sample buffer (63 mM Tris, 10% glycerol, 10% sodium dodecyl sulphate, 5% bromophenol blue) containing 5% 2-mercaptoethano^13,14^ and incubated for 10 min at room temperature. Subsequently, the supernatant was collected by centrifugation at 10,000 × g for 10 min and boiled at 100 °C for 3 min. The prepared samples were electrophoresed on 10% SDS-PAGE and then transferred to polyvinylidene fluoride membranes (Amersham, Piscataway, NJ, USA). Additionally, the SDS-PAGE gel was stained using Coomassie blue to confirm whether the total protein amounts loaded per lane were similar. The polyvinylidene fluoride membranes were then blocked with a blocking agent (3% ECL prime blocking agent, Amersham) for 3 h and then washed twice with PBS-T for 2 min. Subsequently, the membrane was incubated with primary antibody overnight at 4 °C. The primary antibodies used in the present experiment were anti-phospho-PKA substrate (1: 2000, Cell Signaling, Danvers, MA, USA), anti-phosphotyrosine (1: 2000, ab16389, Abcam), and anti-nitrotyrosine (1:1000, ab42789, Abcam). For the internal control, α-tubulin was detected using anti-alpha tubulin (1:10000, ab7291, Abcam). After overnight incubation with the primary antibodies, the membranes were washed with PBS-T and incubated with horseradish peroxidase-conjugated secondary antibody for 2 h. The protein expression was detected using the ECL detection reagent (Amersham) in a dark room. The protein bands were scanned using a GS-800 calibrated imaging densitometer (Bio-Rad, Hercules, CA, USA), and the density of each band was analysed using the Quantity-One 1-D analysis program (Bio-Rad). The data were presented as the ratio of the target protein to α-tubulin (in each lane) in the treated and control samples. For each of the three independent replicate experiments, three male mice were considered for the final results.

### *In vitro* fertilisation (IVF)

For IVF, 10–12-week-old B6D2F1/CrljOri hybrid female mice were purchased from OrientBio^®^ (Gapyeong, Gyeonggi-do, Korea). These mice were intraperitoneally injected with pregnant mare serum gonadotrophin (5 IU) and human chorionic gonadotrophin (hCG; 5 IU) at 48 h intervals to induce superovulation^6,14^. Fifteen hours after the hCG injection, the cumulus-oocyte complexes were collected and transferred to the BM supplemented with 10% fetal bovine serum under mineral oil and incubated at 37 °C for 1 h in 5% CO2 in air. For each treatment, we used approximately 30 oocytes that were collected from at least three superovulated female mice. The collected eggs were pooled and then divided into five groups before being inseminated with the sperm. Simultaneously, the spermatozoa collected from three male mice (for a single experimental replicate) were pooled and divided into five groups that were incubated in BM under various exposure schemes. After incubation, the spermatozoa were washed with 0.4% BSA-containing BM, and 1 × 10^6^/mL spermatozoa were gently inseminated into the oocytes, which were then incubated at 37 °C for 6 h in 5% CO2 in air, as described previously^6,14^. After fertilisation, the normal zygotes were transferred to a fresh BM drop containing 0.4% BSA. The fertilisation rate was evaluated by counting the number of two-cell embryos (cleavage) at 24 h after fertilisation. Every two-cell embryo was again transferred to a fresh BM drop containing 0.4% BSA. Five days later, the numbers of blastocysts were counted. The percentage of cleavage or blastocysts was calculated as the number of cleavage or blastocysts/total single cells used in each treated condition. The final results are representative of four independent experiments.

### Statistical analysis

The data were analysed using one-way analysis of variance (ANOVA) in SPSS Version 23.0 (Chicago, IL, USA), and Tukey’s HSD test was conducted to determine significant differences among the different treatment groups. A *P*-value < 0.05 was considered statistically significantly different. The numerical values represented in this manuscript are mean ± standard error of the mean (SEM).

## Supplementary information

Supplemental Material
