# Supplementary material for: Effect of antioxidants on BPA-induced stress on sperm function in a mouse model
Source: Sci Rep. 2019 Jul 22;9:10584. doi: 10.1038/s41598-019-47158-9 (PMC6646364; doi:10.1038/s41598-019-47158-9)
Supplement: Supplementary file 1 — Supplemental Material [file 41598_2019_47158_MOESM1_ESM.pdf]

SUPPLEMENTAL MATERIAL

Effect of antioxidants on BPA-induced stress on sperm function in a mouse model

Md Saidur Rahman, Kyu-Ho Kang, Sarder Arifuzzaman, Won-Ki Pang, Do-Yeal Ryu, Won-Hee Song, Yoo-Jin Park, and Myung-Geol Pang*

Department of Animal Science & Technology and BET Research Institute, Chung-Ang University, Anseong, Gyeonggi-do 456-756, Republic of Korea.

*Correspondence E-mail: mgpang@cau.ac.kr; Tel: +82.31.670.4841; Fax: +82.31.675.9001

Running title: Antioxidants minimise BPA-induced stress

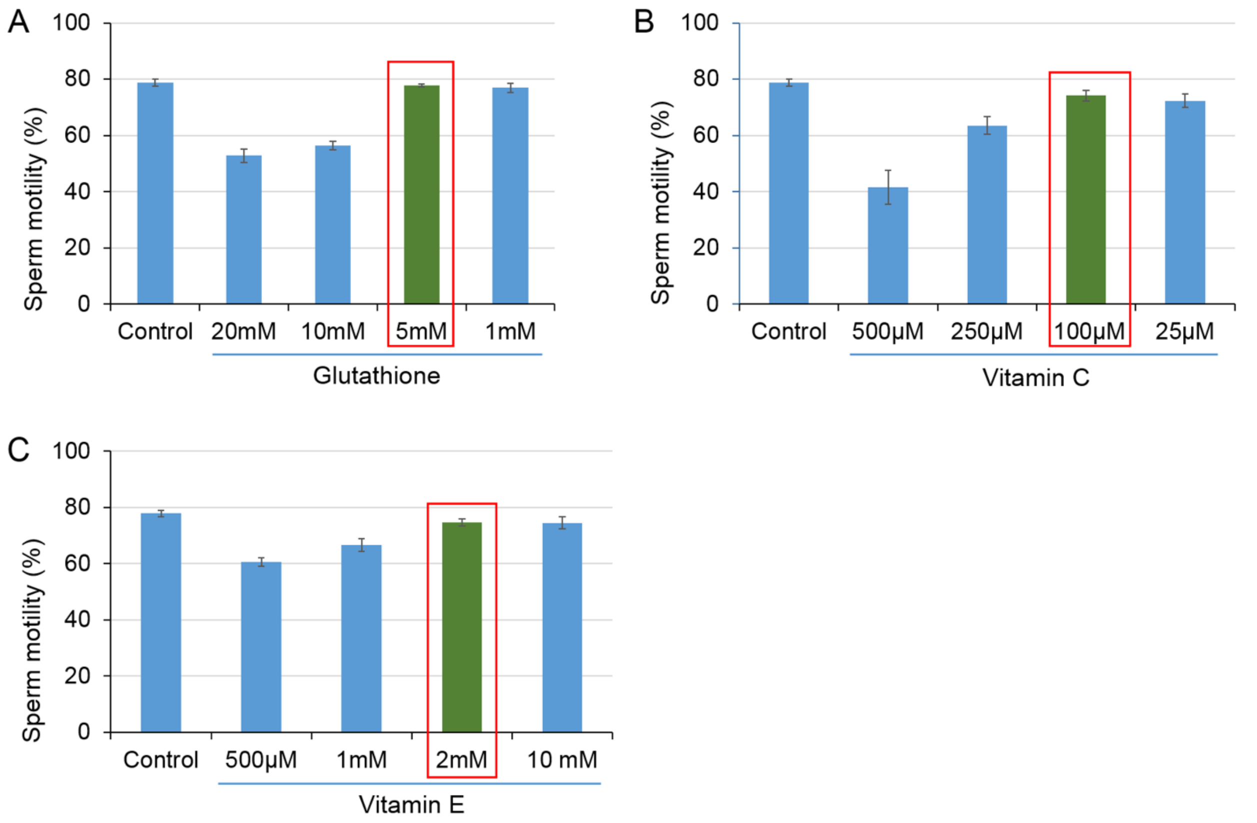

Figure S1. Effects of antioxidant supplementation for 6 h on the motility parameters of spermatozoa. (A) Percentage of motile spermatozoa following exposure to different concentrations of glutathione. (B) Percentage of motile spermatozoa following exposure to different concentrations of Vitamin C. (C) Percentage of motile spermatozoa following exposure to different concentrations of Vitamin E.

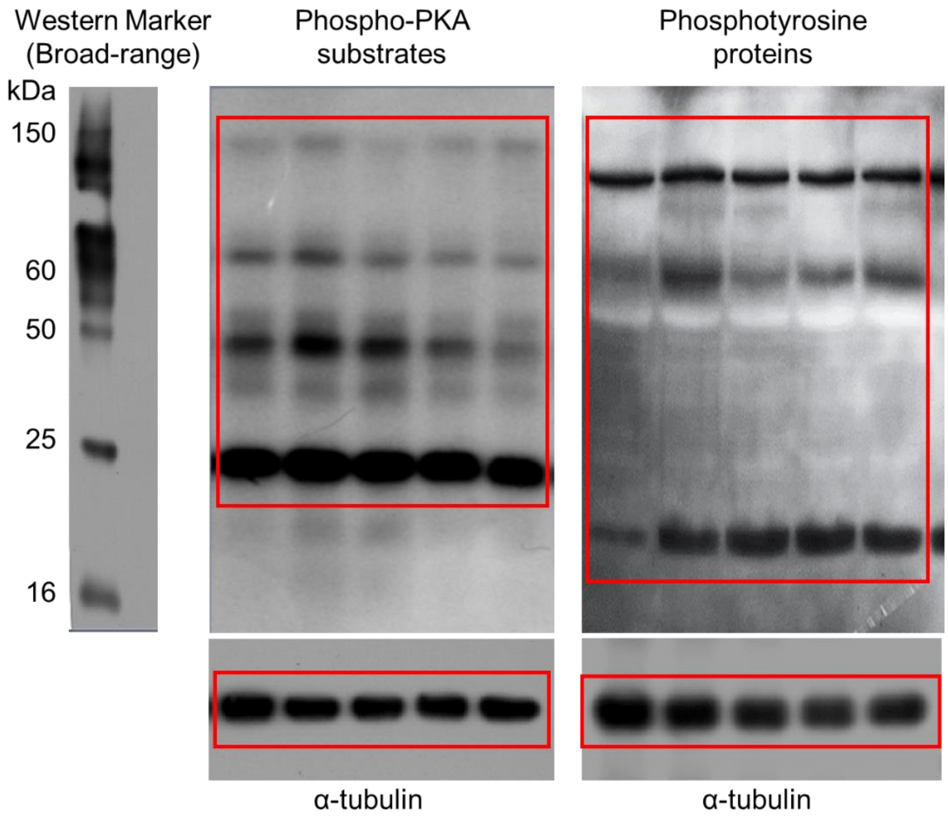

Figure S2. Uncropped images of western blots represented in main Figure 2.

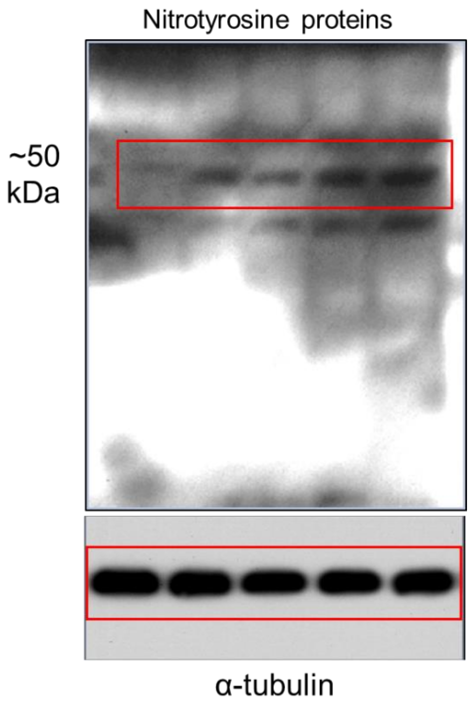

Figure S3. Uncropped images of western blots represented in main Figure 3.
